# Towards User-Oriented Recommendations for Local Therapy of Leg and Foot Ulcers—An Update of a S3-German Guideline

**DOI:** 10.3390/medsci9030054

**Published:** 2021-08-11

**Authors:** Marion Burckhardt, Brigitte Nink-Grebe, Andreas Maier-Hasselmann

**Affiliations:** 1 Angewandte Gesundheits-und Pflegwissenschaften, Campus Horb, Baden-Wuerttemberg Cooperative State University (DHBW), Rotebühlstraße 133, 70197 Stuttgart, Germany; 2 German Association for Wound Healing and Wound Treatment e.V. (DGfW), Glaubrechtstraße 7, 35392 Gießen, Germany; dgfw@dgfw.de; 3 Departement of Vascular Surgery, Munich Municipal Hospital Group, Englschalkinger Str. 77, 81925 München, Germany; andreas.maier-hasselmann@muenchen-klinik.de

**Keywords:** guideline, leg ulcer, foot ulcer, update, methodological standards, protocol, living guideline, wound therapy

## Abstract

Background: The German S3- guideline on local therapy of leg ulcers and diabetic foot ulcers is in the process of being updated. Major goals are to improve the guidelines’ applicability and to take steps towards a living guideline according to current methodological standards. The aim of this article is to describe the main measures to achieve these goals. Methods: The context of the guideline in the field of local wound care and the stakeholder requirements are briefly described. Based on a derived framework, the project team adjusted the methods for the guideline. Results: Main adjustments are more specific inclusion criteria, online consensus meetings and the use of an authoring and publication platform to provide information in a multi-layered format. A new set of practice-oriented key questions were defined by the guideline panel to foster the formulation of action-oriented recommendations. Conclusions: The set of new key questions addressing practical problems and patients’ preferences as well as the adjustments made to improve not only the guidelines’ applicability, but also the feasibility of the further dynamic updating processes in the sense of a living guideline, should be steps in the right direction.

## 1. Introduction

The German guideline “Local Treatment of Chronic Wounds in Patients with Peripheral Vascular Disease, Chronic Venous Insufficiency, and Diabetes” was first published in 2012. The guideline is currently undergoing a fundamental updating process to improve its applicability and user-friendliness while initiating necessary steps towards a living guideline.

The guideline concentrates on local therapy of chronic leg ulcers of either vascular or diabetic origin and complements further national and international guidelines aiming at prevention and causal therapy [1,2]. It aims to present a treatment algorithm, based on evidence and consensus, to optimize local wound therapy in patients diagnosed with peripheral arterial disease (PAOD), diabetes mellitus (DM), or chronic venous insufficiency (CVI). The target groups are health professionals, patients, relatives involved in wound treatment, and other professional groups participating in all areas of care (i.e., physiotherapists). The creation of this guideline is sponsored and coordinated by the German Association for Wound Healing and Wound Treatment (DGfW e.V.) in collaboration with more than 15 societies belonging to the Association of Scientific Medical Societies in Germany (AWMF), as well as patient representatives. Like most of the guidelines which are developed in cooperation with the AWMF and its member medical societies, the guideline is published in German and adapted to the country’s own circumstances in patient care. It can be easily found in the AWMF Guideline Register (www.awmf.org, accessed on 2 August 2021) along with complementary guidelines which focus on the prevention and treatment of the underlying disease as well as concomitant diseases and syndromes whose treatments are a prerequisite for local wound therapy.

In Germany, most guidelines are financed by scientific, medical organizations, rather than national government funds. This means that guideline teams usually have limited resources and most of the work is done by volunteer experts.

Due to the complexity of the topic on the one hand, and differing opinions about the guidelines’ scope on the other, it took years for the first version of this guideline to go from initial ideas to final publishing. In addition, the implementation of high methodological standards for guideline development [3] costs time and resources. However, in the field of “chronic wounds”, guidelines of high validity are necessary, since the topic has for years faced challenges of low evidence, information distortion, and strong commercialization [4]. Therefore, the goal was to create a guideline that is as trustworthy as possible. A stringent project management in line with the “Appraisal of Guidelines for Research and Evaluation (AGREE) Instrument” [3], experienced methodologists, and a range of professional experts have helped to achieve this goal [5].

In the first years after publishing the guideline, its applicability had not been systematically evaluated. The reasons for this were limited resources and a simple lack of “voluntary energy”. However, feedback from health professionals involved in patient care indicated that the guideline was difficult to implement. This was mainly attributed to the complexity of the written text. Moreover, the concerted recommendations and statements themselves seemed to be difficult to transfer into practice. A guideline group that was involved in implementation processes in hospitals became particularly aware of this. In the absence of good evidence for the use of most cleansing solutions and dressings, the guideline group formulated a lot of conservative statements indicating that problem. Many of the recommendations on the other hand were formulated vaguely with few concrete recommendations for action. More than half of all randomized controlled trials (RCTs) investigating wound dressings, topical agents, and accompanying physical measures, which have been identified through our systematic searches covering the last 9 years (2011–2020), included less than 100 participants. Overviews of systematic reviews show no improvement in the quality of evidence either [6,7]. The usability of this evidence for high-grade recommendations is thus limited, and it remains challenging to formulate action-oriented recommendations. For guideline teams in this field, this means that there is a lot of work to meet international methodological standards by reviewing the available evidence, especially if the guidelines are to be up to date.

The project team therefore had two major goals: improving the guideline applicability for its users, and establishing measures to improve the feasibility of the development process to initiate the steps towards a living guideline with high validity.

The aim of this article is to describe the main measures to achieve these goals. These methods are presented in short, and for the purpose of a better understanding, argumentatively reasoned beforehand. The context of the guidelines in the field of local wound therapy will be briefly explained regarding the reasons of low evidence and its consequences for guideline development. Then, the preparatory work to identify potential areas for improvement is described briefly. The problem areas identified here were the basis of major methodological amendments, in particular a newly defined set of key questions. The guideline development process is reported in the guideline report [5]. Any details of modifications, evidence synthesis, and recommendation processes will be published with the updated version at the end of the year 2021.

## 2. The Challenge of Defining Trustworthy Recommendations in the Absence of Trustworthy Evidence

The first version of the guideline was developed in accordance with generally recognized quality criteria as defined in the AGREE instrument [3].

However, implementing high quality standards in a field of low-quality evidence proved to be a challenge. This was mostly due to the fact that it is not easy to formulate trustworthy recommendations in the absence of trustworthy evidence.

Noninvasive medical devices, to which most wound treatment products belong, only have to prove their safety and intended purpose before they can be launched on the market. This means that the approval of a wound dressing for example, requires evidence that it absorbs exudate or helps to maintain a moist wound environment. It does not require proof of efficacy on the basis of RCTs in outcomes like “wound healing” or “pain”, which are relevant for patients.

By comparison, in the pharmaceutical sector, this proof of efficacy is essential for more than fifty years [8,9]. If such studies are available in wound care and are well conducted, it can be judged whether improved wound healing is a coincidence or whether it is the result of the wound dressing.

Since RCTs are not required for the market approval of wound treatment products, these expensive and time-consuming studies are often not carried out at all or are only presented at conferences rather than being published in scientific journals [10]. The majority of published RCTs are judged with insufficient reporting quality or high risk of bias. They further lack sufficient sample size, fail to define primary outcomes or use surrogate measures instead of outcomes relevant for patients (like morbidity) [11].

Although well-conducted RCTs are possible within the field of wound therapy (i.e., [12]), they are very rare. The overall quality of the evidence can be judged with the GRADE approach by considering criteria like inconsistency of the results, risk of bias, and other criteria which can influence the effect estimates. These quality ratings (which can go from “very low” to “high”) give a good impression of the extent of our confidence that the estimates of an effect are adequate to support a particular decision or recommendation” [13]. In the field of wound healing, the problem of low or even absent evidence becomes apparent in recent Cochrane Reviews. In those investigating wound dressings and adjuvant wound therapies (i.e., negative pressure) within the scope of the guideline, the quality of evidence was judged, at best, as “low” for all outcomes relevant for the patients [6,7,14,15,16,17,18,19].

## 3. Preparatory Work to Identify Potential for Improvement

In preparation for the update process, we assessed the need for improvement with a critical quality analysis of the guideline documents [5,20,21]. We also did a stakeholder survey to identify necessary improvement areas related to applicability and user expectations. The details of the methods and results are reported elsewhere [22]. To illustrate the relationship with update measures that have been initiated, they are briefly summarized.

To identify potential for improvement of our methods, the original guideline was critically appraised by two independent experts, according to the AGREE-II-Instrument. The AGREE-II instrument [3] not only assesses methodological rigour (from evidence synthesis to reasonable recommendation), but also helps to identify areas which can influence implementation. These are, for example, the involvement of representative stakeholders, the methods to control possible conflicts of interest, and recommendations to improve applicability and clarity in the guideline presentation.

External assessments of the methodological quality have also been taken into account, like a structured assessment of conflicts of interests from the group “guideline watch” [23] and peer reviews from medical and experts and scientists [22]. Altogether, the methodological rigor was assessed as high. Potential for improvement was mainly seen in areas in which higher standards have been developed within the last ten years. Conflicts of interest, for example, have been reported, and the voting during the consensus processes was controlled for individual- and group-psychological influencing factors [5]. This can be achieved by using formal, independently moderated consensus techniques [24]. Standards developed in the last few years [25] call for stricter rules controlling for conflicts of interest (i.e., by reporting decisions transparent to each recommendation). The reporting of the guideline was also transparent, but could be improved if the search strategies were fully reported according to the PRISMA reporting standard [26]. The guideline team also used the GRADE approach to assess the quality of the evidence for each outcome, but did not prioritize them as suggested by the GRADE working group [27]. One of the external peer reviewers commented that the guideline does not address practical questions like “how and how often should a wound be cleaned?” The guideline included only PICO questions like “what is the effect of a cleansing solution X in patients within the scope of the guideline?”

For the stakeholder survey [22], health care providers, organizations, and individual health care personnel involved in wound care in Germany were contacted by email and internet and provided with a short questionnaire. The open questions addressed the necessary amendments and the new topics based on the stakeholders’ experience in inpatient and outpatient care, as well as the applicability of the guideline. Suggestions came mainly from health care providers in leading positions. These were summarized thematically and gave valuable insight to the practical relevance of the guideline. Apart from a few suggestions relating to expanding the scope of the guideline (i.e., with respect to the inclusion of surgical interventions and infected wounds), the main problem identified was in the applicability in practice. In short, the users wanted action-oriented recommendations even in the absence of evidence. The low practical value of a statement indicating insufficient evidence for an expert recommendation was criticized. Instead, the stakeholders wanted concrete instructions for actions in everyday wound care or at least corridors for such actions. Furthermore, it was suggested that teaching materials should be provided to healthcare facilities to help implement these guidelines [22].

It was obvious that there was an imbalance between the highly assessed methodological rigor and transparent reporting on the one hand, and the readability for health professionals on the other hand.

**Figure 1:**
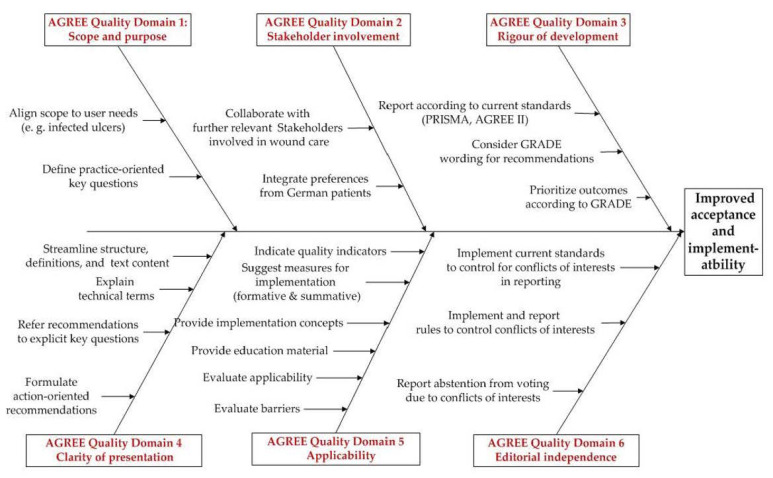
Framework to improve acceptance of the German DGfW-guideline (translated from [22]).

The quality of a guideline as described in the AGREE II-Instrument and its implementability are strongly correlated. Therefore, it can not only be used to assess a guidelines’ validity and reporting, but with its six domains, it also provides a strategy to develop guidelines [3]. The stakeholders’ suggestions to improve the applicability, the results of the critical appraisal with AGREE II, the external evaluations and comments aiming at improvement as submitted by peer reviewers were then synthesized according to the six AGREE domains and presented in a cause-and-effect diagram to provide a framework for further planning (Figure 1).

Considering the situation described above, the available resources for reviewing the evidence, and that the guidelines should be constantly updated (in the sense of a “living guideline”) the guideline team decided on the following methodological adjustments for the update:

## 4. Measures to Increase User-Friendliness

The first version of these guidelines was more than 250 pages long and included detailed summaries, written by more than thirty authors in varying degrees of detail. For the updated version, all chapters will be abridged and prepared by an experienced guideline author in a structured format. An electronic version will be uploaded on an authoring and publication platform called MAGIC (MAGICapp) [28] to provide information in a multi-layered format. This will allow users to decide how much background information is needed. A digital platform further enables dynamic updates of certain chapters if the evidence leads to a change of some recommendations. Additional implementation and education material will be prepared.

The original guideline version included goal-oriented algorithms. They clearly describe the process from first diagnostic assessment to treatment, while also referring to other guidelines related to prevention and causal therapy. English versions of these are presented in [29]. These algorithms will be maintained and adjusted to the new recommendations.

A core component of the guideline update is a new set of key questions, which were agreed on by the panel in a formal moderated session. These questions address practical questions to foster the formulation of action-oriented recommendations. The key questions comprise a range of topics including initial assessment, treatments for different wound symptoms, and organizational issues (Table 1). The panel also judged the following outcomes as critical for recommendations according to the GRADE approach [30]: complete epithelization of the wound, time to wound healing, serious adverse events, wound infection, and pain (related to wound or treatment).

## 5. Methods to Fulfill AGREE Requirements and Steps towards a Feasible Living Guideline

### 5.1. Involvement of Stakeholders and Patients

The guideline panel came from multiple health disciplines involved in wound therapy. Scientific medical societies within the AWMF appointed representatives with voting rights for final recommendations. Conflicts of interests are handled according to current AWMF standards [25].

Patient preferences in wound care were considered in several steps. In preparation of the first version of the guideline in 2012, preferences were identified using a systematic approach. Preferences were synthesized from qualitative studies, surveys, a patient charter and verified with directly affected patients and an official representative appointed from a self-help organization in Germany. The main preferences of patients suffering from wounds within the scope of the guideline referred to:

- Optimal cooperation with the therapeutic team;
- Good organization of care;
- High quality of medical-technical care administered by all participating health professions [5].

Keeping these requirements in mind, the key questions address several topics related to documentation, organization of care, education of personnel, and interaction with patients (Table 1).

### 5.2. Inclusion Criteria and Search Methods

Whereas the first version even included pilot studies, future releases will use more specific and quality guided criteria to accelerate the synthesis process:

- Cochrane Systematic Reviews or other systematic reviews with at least moderate quality (assessed with AMSTAR [30]);
- Additional RCTs within the scope of the guideline are only considered if they have a sufficient sample size (operationalized as at least 100 participants).

In view of the low quality of evidence for interventions in question (as indicated in most Cochrane reviews published in the last years), it is not expected that single studies with small sample sizes would change the confidence enough to have a significant impact on the grading of recommendations. The search was limited to published English and German articles considering patients, interventions, and outcomes within the scope of the guideline. The full search strategy is published with the former guideline report [5].

### 5.3. Appraisal of Strengths and Limitations of the Evidence

The validity of systematic reviews was assessed with AMSTAR [31]. Cochrane Reviews were not appraised with AMSTAR, since they follow broadly agreed methodological standards and a thorough peer review process [32] so that moderate quality can be expected. We assessed the quality of the evidence based on the GRADE approach [13]. If available, we used the GRADE rating as judged by the authors of systematic reviews. We carried out double checks on the assessments on a selection of studies and offered the evidence synthesis to the whole guideline team to review the results of the evidence synthesis. Disagreements were solved in discussion within the team.

### 5.4. Formulation of Recommendations

First, all recommendations from the former guideline version were reviewed by the guideline group (all authors and representatives from collaborating organizations were invited). They considered the need for revision in view of new evidence and practice problems, as well as the new set of key questions. All suggestions for newly formulated or adapted recommendations were justified with a short rationale within the team. These informal meetings were initially carried out virtually in winter and spring 2020/21. To respect the limited time resources of the experts and to avoid tedious discussions with the danger of unwanted majority influences, all sessions were limited to one hour. When few experts attended the sessions, or if opinions were quite different, we presented suggestions for revisions of recommendations again in other meetings.

In a second step, which should be completed by June 2021, the guideline coordination team will check all recommendations for consistency in the wording, particularly in view of the existing evidence. All recommendations will be summarized with a rationale following the GRADE evidence to decision framework [33] by considering benefits, harms, and the quality of evidence. If necessary, to justify a recommendation, further aspects like feasibility, acceptability, or resource use will be considered too. These drafts will be sent to all authors and representatives of AWMF medical societies for review.

Formal (virtual) consensus meetings are set in summer 2021. They will be moderated by an AMFW-guideline commissioner, with formal consensus methods controlling for conflicts of interests of representatives entitled to vote. This process will follow the standards of the AWMF [25]. Based on conflicts of interests of all panel members, reviewed beforehand, voting modalities will be formulated in advance and transparently described within the guideline report for each key question.

In the last step, the edited guideline and related documents will be sent to all collaborating organizations for final official agreement. All methods will be published in an amendment to the guideline.

## 6. Discussion

Local wound therapy is characterized by a high variety of cleansing solutions, dressings, and adjuvant interventions but a low quality of evidence. It is a challenge for a guideline panel in this field to formulate trustworthy recommendations. A stakeholder analysis and critical appraisal of the former version of the guideline indicated that there is a need for action-oriented recommendations addressing questions of the health professionals in practice. Therefore, the guideline underwent a fundamental revision during the update process. Major goals were guideline applicability and user-friendliness. Furthermore, a new set of key questions have been formulated in order to foster action-oriented recommendations to the guideline panel. However, it is a prerequisite that a consensus of the representatives on these recommendations can be achieved, in view of the low quality of evidence.

Ensuring a high methodological quality, in view of numerous studies with insufficient sample sizes and high risk of bias on the one hand and limited time resources on the other hand, leads to further adjustments. There are still some limitations. It is possible that even small RCTs provide a high certainty of evidence for an intervention, but it can be assumed that these would be integrated quickly in systematic reviews. Providing high-quality instructional materials will be another challenge. Guidelines in Germany are not supported by national funds and guideline developers are forced to find the best balance between high methodological quality, applicability, and feasibility. National funds to support the evidence synthesis, dissemination into practice, and evaluation of the recommendations’ implementation are rare in Germany [34] and should be discussed more with decision-makers.

Nevertheless, many organizations like the German AWMF with its member societies, AGRRE Enterprise, the GRADE working group, and the MAGIC-team provide support, create quality standards or method handbooks, and develop digital solutions and decision frameworks. The development of guidelines would also be simply impossible without the work of numerous experts and researchers volunteering their support through discussing content, reviewing the extensive evidence, formulating recommendations, or providing high quality evidence syntheses like Cochrane reviews provided by the Cochrane wounds group.

This comprehensive preliminary work and ongoing support promotes the development of a trustworthy guideline, which is necessary in a field of high practice variability and great uncertainty in terms of efficacy. However, even the best guideline will not have a significant impact on patients’ care if it is not implemented in a comprehensive way. The set of new key questions addressing practical problems and patients’ preferences as well as the adjustments made to improve not only the guidelines’ applicability, but also the feasibility of further updating processes in the sense of a living guideline should be steps in the right direction.

## Figures and Tables

**Table 1:** Key guideline questions for local therapy of wounds in leg ulcers and diabetic foot ulcers.

Key Questions
What are the key parameters medical doctors should collect as a basis for decisions within clinical examination and diagnostics? At which point would additional diagnostic testing and/or specialist referral be considered appropriate or necessary? What topics should medical advice to affected patients cover? What are the key parameters health care professionals should collect within (wound-) assessment as a basis for decisions? When should a formal clinical wound-assessment be performed? What are the minimum parameters that should be included in a formal clinical wound assessment? At what point should active wound cleansing 1 be done? How should a wound with no signs of infection and no signs of slough be cleaned? How should a wound with clinical signs of infection be cleaned? How should a wound with slough, eschar, or necrotic tissue present in the wound bed be cleaned? At what point should additional “passive cleansing measures” 2 be added to a standard wound cleansing regimen? Which “passive cleansing measures” 2 should be taken in addition to a standard wound cleansing regimen? Under which conditions does a wound require covering? How should a wound with no signs of infection be managed, and what kind of wound dressing is appropriate? How should a wound with signs of infection be managed and what kind of wound dressing is appropriate? Which criteria should be taken into account when choosing an appropriate wound dressing? Which dressings are appropriate for painful wounds? Which dressings are appropriate for wounds with odor? How should surrounding tissue and skin be cared for and protected? Which physical measures should be used supplementary? How should particularly large or deep wounds be managed? What is the appropriate procedure in a wound that, despite proper causal and local therapy, stagnates in its healing? How should chronic wounds be handled in daily life (i.e., with respect to bathing or showering) Under which conditions, in the context of ongoing treatment of a chronic leg ulcer, should allergy-testing be considered? How should healed ulcers be treated? How should patient care be organized? Which topics should be addressed by qualification measures within the scope of the guideline?

^1^ Defined as wound cleansing during dressing change. ^2^ Defined as wound cleansing measures between dressing changes (i.e., larval therapy).

## Data Availability

The data to the guideline (i.e., systematic searches, quality appraisal, conflicts of interest of all involved persons) are openly available at the AWMF website: https://www.awmf.org/leitlinien/detail/ll/091-001.html (accessed on 2 August 2021).
